# Adsorption Behavior of Fe(II) and Fe(III) Ions on Thiourea Cross-Linked Chitosan with Fe(III) as Template

**DOI:** 10.3390/molecules17044388

**Published:** 2012-04-11

**Authors:** Jun Dai, FengLian Ren, ChunYuan Tao

**Affiliations:** 1 College of Chemistry and Chemical Engineering, Central South University, Changsha 410083, China; Email: deardaijun@sina.com; 2 College of Chemistry and Chemical Engineering, JiuJiang University, JiuJiang 332005, China; Email: taochunyuan@sina.com

**Keywords:** adsorption, thiourea, cross-linked chitosan, template, iron ion

## Abstract

A new type of thiourea cross-linked chitosan with Fe(III) as template (TCCTS template) was synthesized. The adsorption of Fe(II) and Fe(III) on this TCCTS template was studied. The factors affecting adsorption such as pH and contact time were considered. The results showed that the optimum pH value for adsorption was pH = 5.0 and the adsorption equilibrium time was about 60 min. The adsorption isotherms and kinetics were investigated, and the equilibrium data agreed very well with the Langmuir model and the pseudo second-order model could describe adsorption process better than the pseudo first-order model. Results also showed that TCCTS template was a favourable adsorbent for Fe(II) and Fe(III) in aqueous solution.

## 1. Introduction

Water contamination by heavy metal ions is a serious environmental problem. Heavy metals can be toxic pollutants that are nonbiodegradable, and have environmental, public health and economic impacts [1]. Iron is a major one of these heavy metals, it is the second most abundant metal element in the Earth^,^s crust, and is mainly present in natural water as two oxidation states: Fe(II) and Fe(III). Fe(II) is essential for proper transport and storage of oxygen by means of hemoglobin and myoglobin while its oxidized forms, methemoglobin and metmyoglobin, which contain Fe(III), will not bind oxygen [2]. Iron is very important in the biosphere, it plays a essential role in photosynthesis and is the limiting growth nutrient for phytoplankton in some parts of the ocean [3]. Iron in environmental water comes from steel tempering, coal coking and mining industries [4]. 

The methods used for the removal of trace metals from water include chemical precipitation [5], ion exchange [6], solvent extraction [7] and adsorption [8]. Among these methods, adsorption has been proved to be an efficient and economical technique. Activated carbon and silica gel are the two most popular adsorbents [9] in trace element analysis. But they are relatively expensive materials since the higher the quality, the greater their cost. Looking for alternative adsorbents has intensified in recent years. At present, the focus is on chitosan. Chitosan is prepared from chitin by deacetylating its acetamido groups to a different degree. Chitosan has both hydroxyl and amine groups that can be chemically modified [10,11,12], by reactions such as cross-linking, grafting, alkylation and esterification. Chemically modified chitosan has been proved to be highly efficient at removing heavy metal ions from dilute solutions [13].

Because cross-linkers like glutaraldehyde [14] and epichlorohydrin [15] would weaken the adsorption efficiency of chitosan, the insertion of cross-linking agent with functional groups into chitosan is an effective way to get adsorbents with good adsorption capacity. Thiourea has been proved to be a good material to modify chitosan and improve its adsorption ability for many heavy metals [16,17]. In this work, we synthesized thiourea cross-linked chitosan with Fe(III) as template. We attempted not only to enhance the adsorption ability, but also to improve the selectivity of chitosan by doing this. The adsorption behavior of Fe(II) and Fe(III) on the TCCTS template was investigated and the adsorption isotherm and kinetics were studied.

## 2. Results and Discussion

### 2.1. FTIR Analysis

**Figure 1:**
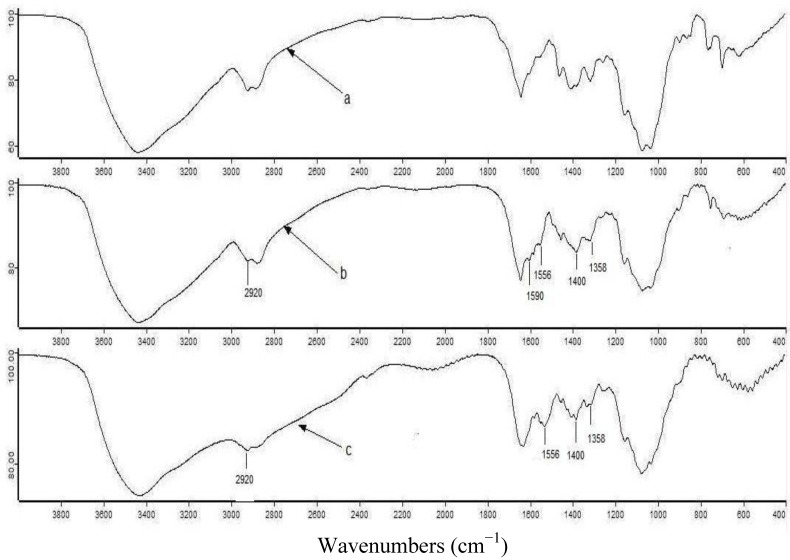
IR spectra of CTS (**a**), TCCTS template before (**b**) and after (**c**) Fe(III) removal.

The FTIR spectra of CTS, TCCTS template before and after Fe(III) removal are presented in Figure 1. As compared with CTS, some new bands appeared in the spectrum of the TCCTS template. The new band near 1,556 cm^−1^ is the vibration adsorption peak of the thiourea moiety C–N group. The characteristic adsorption peak of thiourea, which is assigned to the N–C=S group, appears at about 1,400 cm^−1^ [18]. Moreover, obvious changes in the spectra of TCCTS template before and after Fe(III) removal were observed. The new band around 1,590 cm^−1^ in the spectrum of TCCTS template before Fe(III) removal disappeared in the spectrum of TCCTS template after Fe(III) removal, at the same time, the intensity of the band at 1,358 cm^−1^ was increased in the spectrum of TCCTS template after Fe(III) removal. This indicated that Fe(III) was removed in the final step of the synthesis process.

### 2.2. Characterization by SEM

**Figure 2:**
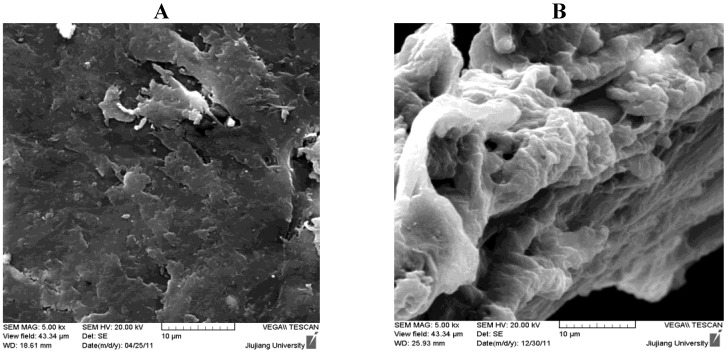
SEM of (**A**) CTS and (**B**) TCCTS template.

Figure 2 shows the SEM pictures of CTS and TCCTS template. From which we could see that the surface of CTS was relatively smooth and the structure of CTS was compact. The surface of TCCTS template was rough, there were a lot of cavities in its structure and the structure was reticular and incompact. This three dimensional structure of TCCTS template is favorable for metal cation adsorption [19].

### 2.3. Effect of pH on Adsorption of Fe(II) and Fe(III)

**Figure 3:**
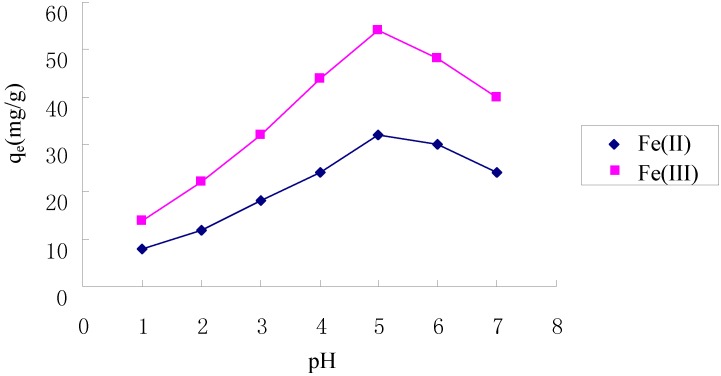
Effect of pH on adsorption of Fe(II) and Fe(III).

As shown in Figure 3, the adsorption of Fe(II) and Fe(III) increased with increasing pH when pH < 5.0. Chitosan and its derivatives adsorb metal cations mainly by chelation with amine groups [20], at lower pH, the adsorption of Fe(II) and Fe(III) decreased because some amine groups were protonated to form –NH_3_^+^, reducing the number of binding sites available for the adsorption. When the pH value increased, the adsorption of Fe(II) and Fe(III) increased due to the decreasing number of protonated amine groups and the resulting increase in the number of binding sites. The maximum adsorption for both Fe(II) and Fe(III) on the TCCTS template appear at pH 5.0. The adsorption capacity of Fe(III) is higher than that of Fe(II), mainly because the TCCTS template has a memory effect for Fe(III). In the higher pH range, Fe(II) and Fe(III) precipitation occurred, which resulted in the adsorption of Fe(II) and Fe(III) decreasing.

### 2.4. Kinetics of Adsorption

**Figure 4:**
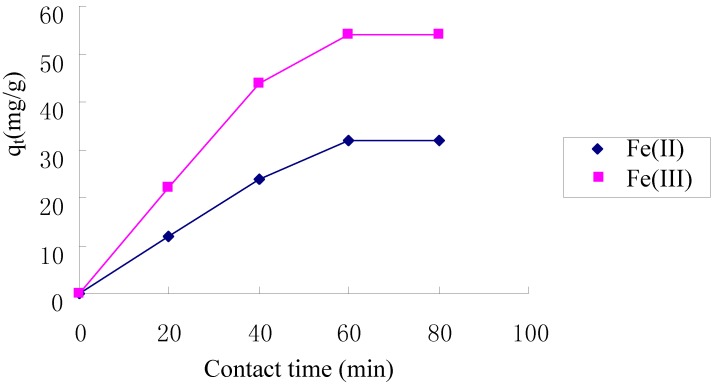
Effect of contact time on adsorption of Fe(II) and Fe(III).

**Figure 5:**
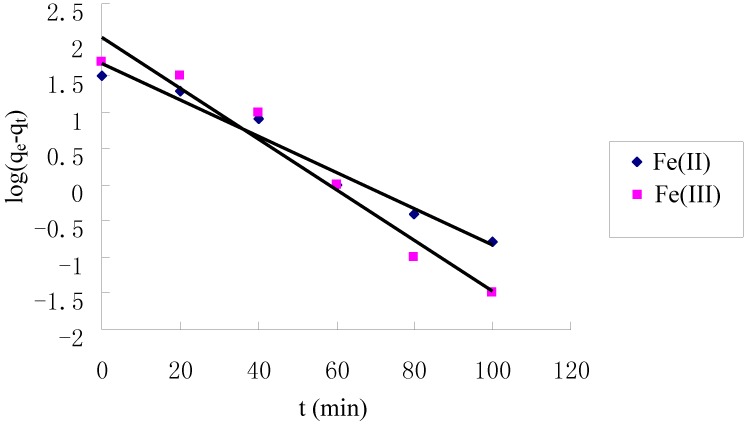
Pseudo first-order kinetic plots for the adsorption of Fe(II) and Fe(III).

**Figure 6:**
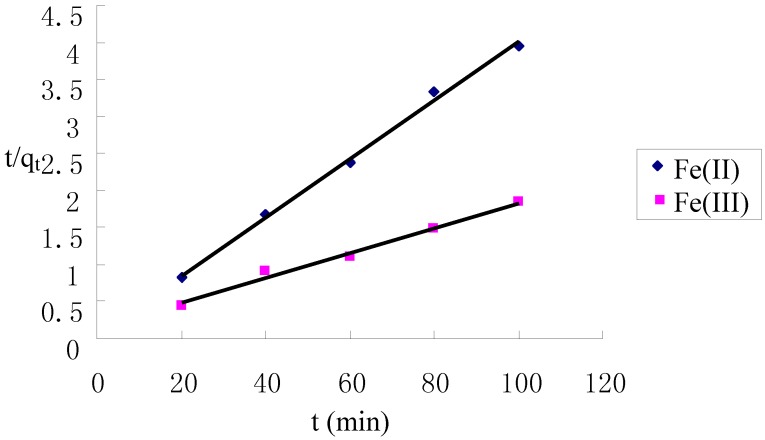
Pseudo second-order kinetic plots for the adsorption of Fe(II) and Fe(III).

Figure 4 shows that the adsorption of Fe(II) and Fe(III) increased with increasing contact time and reached equilibrium at about 60 min on the TCCTS template. In order to investigate the adsorption process kinetics, the pseudo first-order and pseudo second-order kinetic models were applied in this study. The pseudo first-order model is expressed as [21]:


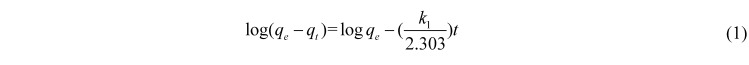


where q_e_ and q_t_ (mg/g) are the amounts of Fe(II) and Fe(III) adsorbed on TCCTS template at equilibrium and at time t, respectively, and k_1_ is the pseudo first-order rate constant (min^−1^) of adsorption. The rate constant, k_1_ and correlation coefficient, R^2^ were determined by plotting the log(q_e_ − q_t_) versus t. The pseudo second-order model is expressed as [22]:


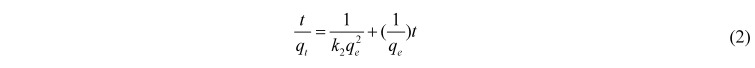


where k_2_ is the pseudo second-order rate constant(g·mg^−1^·min^−1^) of adsorption. The rate constant, k_2_ and correlation coefficient, R^2^ were determined by plotting the t/q_t_ versus t. The kinetic models for Fe(II) and Fe(III) adsorption are shown in Figure 5 and Figure 6. The parameter values of the kinetic models are presented in Table 1. According to Figure 5 and Figure 6 and based on the correlation coefficient in Table 1, the pseudo second-order model could better describe the adsorption of Fe(II) and Fe(III) on the TCCTS template than the pseudo first-order model. This suggests that the rate-limiting step may be chemical adsorption [8]. In many cases, the pseudo second-order model correlates well to the adsorption of metal cation on chitosan and its derivatives [23].

**Table 1:** Kinetic parameters for Fe(II) and Fe(III) adsorption on TCCTS template.

Metal ion	Pseudo first-order		Pseudo second-order	
	k_1_ (min^−1^)	R^2^	k_2_ (g·mg^−1^·min^−1^)	R^2^
Fe(II)	0.058	0.970	0.028	0.996
Fe(III)	0.081	0.963	0.0019	0.990

### 2.5. Adsorption Isotherms

**Figure 7:**
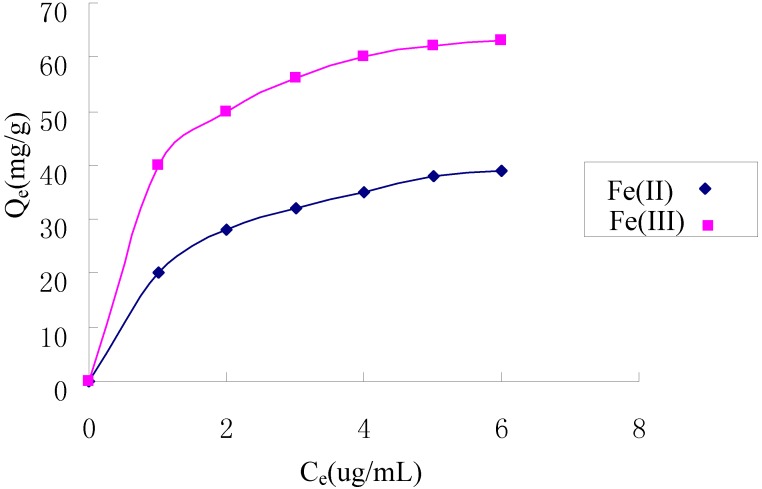
Adsorption isotherms of Fe(II) and Fe(III).

Figure 7 shows the adsorption isotherms of Fe(II) and Fe(III) on the TCCTS template. The Langmuir and Freundlich equations were applied to experimental data in Figure 7 to examine the relation between sorption and metal ion concentration at equilibrium. The Langmuir model, which is widely used for monolayer sorption on a surface, is presented as:


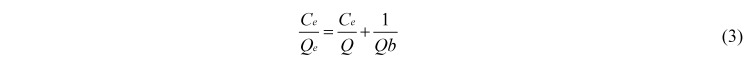


where Q_e_ (mg/g) is the adsorption capacity of Fe(II) and Fe(III) at equilibrium concentration, Q (mg/g) is the maximum adsorption capacity, C_e_ (μg/mL) is the equilibrium concentration of Fe(II) and Fe(III), b (mL/μg) is the Langmuir constant. Q and b can be calculated by plotting C_e_/Q_e_ versus C_e_. For Langmuir model, it is estimated by a dimensionless separation factor whether the sorption is favorable or not. The separation factor, R_L_ is defined as:


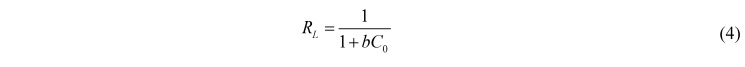


where C_0_ (ug/mL) is the initial concentration of Fe(II) and Fe(III), b (mL/μg) is the Langmuir constant. Values of 0 < R_L_ < 1 indicates that the sorption is favorable. The values of R_L_ in this study lie in the range of 0.15 and 0.42, 0.09 and 0.29 for Fe(II) and Fe(III), respectively, which shows that the adsorption of Fe(II) and Fe(III) on the TCCTS template are favorable. 

The Freundlich model, which is widely used for sorption on a heterogeneous surface, is given by:


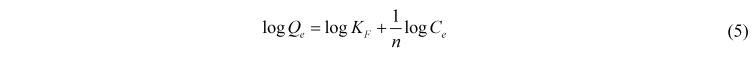


where K_F_ and n are Freundlich constants related to adsorption capacity and intensity, respectively. K_F_ and n can be determined from a linear plot of logQ_e_ versus logC_e_. The constants of the two models along with correlation coefficient (R^2^) values are presented in Table 2. It is found that the Langmuir model fit the data better than the Freundlich model, which indicates that the adsorption of Fe(II) and Fe(III) on the TCCTS template is a type of monolayer sorption.

**Table 2:** Parameters of Langmuir and Freundlich models for Fe(II) and Fe(III) adsorption.

Metal ion	Langmuir model			Pseudo second-order		
	Q (mg/g)	b (mL/ug)	R^2^	K_F_ (mg/g)	n	R^2^
Fe(II)	48.3	0.692	0.9988	20.5	2.52	0.9875
Fe(III)	71.9	1.19	0.9994	40.2	3.53	0.9872

### 2.6. Selective Adsorption of Fe(III)

The adsorption capacity obtained from binary mixtures is presented in Table 3. The data showed that the TCCTS template was effective in selective adsorption of Fe(III) from solutions containing other metal ions.

**Table 3:** Adsorption capacity for Fe(III), Pb(II), Zn(II), Cd(II), Ni(II).

Metal ion	Adsorption capacity (mmol/g)
Fe(III)	1.38
Pb(II)	0.35
Zn(II)	0.46
Cd(II)	0.18
Ni(II)	0.50

## 3. Experimental

### 3.1. General

Chitosan (deacetylation degree 90%) and thiourea were purchased from Shanghai National Reagent Company. The other reagents are all of analytical grade and provided by the chemistry laboratory of JiuJiang University. 0.1 mol/L HCl and 0.1 mol/L NaOH were used to control the pH values of the solutions, 1 g/L Fe(II) and Fe(III) stock solution were prepared by dissolving the appropriate amount of FeCl_2_ and FeCl_3_·6H_2_O in doubly distilled water, which was used throughout the entire experiment. Iron was determined on a FAAS model AA6300C (Shimadzu, Kyoto, Japan) with a iron hollow cathde lamp and a deuterium background correction. Its operating conditions are given in Table 4. pH values was measured on a pH meter model PHS-3C(Shanghai Precision Instrument Company, Shanghai, China). IR spectrum of the product was performed on an infrared spectrometer model Vertex70 (Bruker, Germany) with KBr disc method. The SEM image was performed on a SEM model Vega ΙΙ (Tescan, Czech).

**Table 4:** FAAS operating conditions.

FAAS parameters	
Lamp current(mA)	12
Slit width(nm)	0.2
Flow rate of acetylene(L/min)	2.2
Flow rate of air(L/min)	15.0
Analytical wavelength(nm)	248.3

### 3.2. Preparation of TCCTS Template

**Scheme 1:**
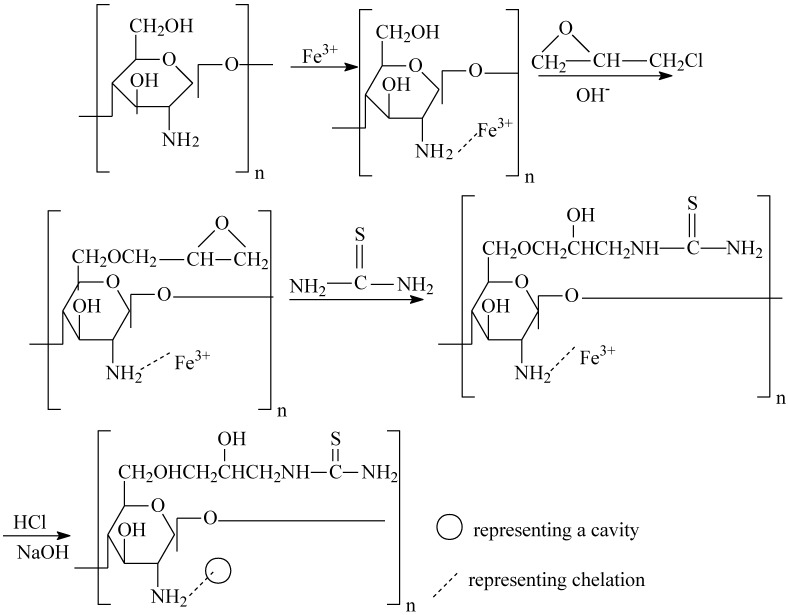
Proposed procedure of synthesis of TCCTS template.

The process of preparation included the following steps (Scheme 1):

(1) CTS (6.0 g) was dissolved in 1% aqueous solution of acetic acid (60 mL) and then added to a 500 mL beaker containing iron trichloride solution (200 mL, 0.02 mol/L). The pH of the solution in beaker was adjusted to 5.5, the beaker was shaken for 24 h at 300 rpm, 25 °C until the product was obtained. The solid product was filtered under reduced pressure. The filtrate was washed with distilled water until no Fe(III) ion was detected by KSCN solution and then the product was dried at 60 °C in vacuum.
(2) The product obtained from step (1) (4.5 g) was dissolved in 5% NaOH solution (100 mL). Epichlorohydrin (15 mL) was added to the solution in a three-necked flask. The mixture was heated on a water bath for 4 h at 70 °C. Thiourea (6.0 g) was dissolved in distilled water (100 mL), and the solution was then added to the mixture of the flask and heated for 5 h at 70 °C until the product was formed. The product was filtered under reduced pressure and washed several times with ethanol followed by distilled water. Subsequently the filtrate was put into a beaker containing 0.1 mol/L HCl (100 mL) and stirred for 2 h at 25 °C, then filtered. This process was repeated until no Fe(III) ion was detected in solution. The filtrate was treated with 0.1 mol/L NaOH solution for 5 h and then washed several times in turn with ethanol, acetone, distilled water. The TCCTS template was obtained after dried at 60 °C in vacuum for 5 h.

### 3.3. Effect of pH

The effect of pH on adsorption of Fe(II) and Fe(III) was studied in pH range 1.0–7.0 at 25 °C by shaking dry TCCTS template (10 mg) with Fe(II) and Fe(III) solution (100 mL, 6 μg/mL) for 60 min at 300 rpm. The desired pH was adjusted using 0.1 mol/L HCl and 0.1 mol/L NaOH. After filtration, the concentration of Fe(II) and Fe(III) in solution was determined by FAAS.

### 3.4. Kinetics of Adsorption

Kinetic studies were conducted by placing TCCTS template (10 mg) in a 250 mL flask containing Fe(II) and Fe(III) solution (100 mL, 6 μg/mL) at pH 5.0 and 25 °C. The mixture was stirred by a magnetic stirrer at 300 rpm. Samples of solution (10 mL) were withdrawn at scheduled time intervals after filtration and analyzed for Fe(II) and Fe(III) concentration.

### 3.5. Adsorption Isotherms

At 25 °C, a series of different concentrations of Fe(II) and Fe(III) standard solutions (100 mL) were prepared. The pH of the solution was adjusted to 5.0. TCCTS template (10 mg) was added into solution and the solution was stirred at 300 rpm for 60 min, then filtered. After filtration, the concentration of Fe(II) and Fe(III) was determined by FAAS. The adsorption capacity was calculated according to:


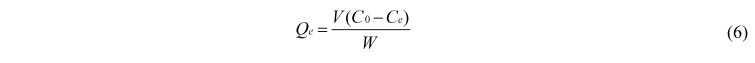


where *Q*_e_ (mg/g) is the adsorption capacity, *C*_0_ (μg/mL) is the initial concentration of Fe(II) and Fe(III), *C*_e_ (μg/mL) is the equilibrium concentration of Fe(II) and Fe(III), *V* (mL) is the volume of the solution of Fe(II) and Fe(III), *W* (mg) is the weight of TCCTS template added.

### 3.6. Selectivity for Adsorption of Fe(III)

The selective adsorption of Fe(III) from binary mixtures with Pb(II), Zn(II), Cd(II), Ni(II) (0.01 mol/L for each metal ion) was carried at pH 5.0. Dry TCCTS template (0.1 g) was added into the binary mixture solution and the other adsorption conditions is the same as described in Section 3.3. After adsorption equilibrium, the concentration of each metal ion was determined by FAAS.

## 4. Conclusions

In this study, the adsorption capacity of Fe(II) and Fe(III) on TCCTS template was examined, and both adsorption equilibrium and adsorption kinetics were investigated. The adsorption isotherms could be well fitted by the Langmuir equation and the adsorption process could be best described by the pseudo second-order kinetic model. The adsorption behaviour of Fe(II) and Fe(III) on the TCCTS template was very similar, but the adsorption capacity of Fe(III) was much greater than that of Fe(II). Both this and selective adsorption experiment indicate that the TCCTS template has relative selectivity for adsorption of Fe(III). According to the result of this study, it can be concluded that the TCCTS template is effective adsorbent for the removal of iron ions from waste water.
